# Development of salutogenic coping skills: experiences with daily challenges among young adults suffering from serious mental illness

**DOI:** 10.1080/17482631.2021.1879369

**Published:** 2021-01-27

**Authors:** Jan-Freddy Hovland, Bente O. Skogvang, Ottar Ness, Eva Langeland

**Affiliations:** a Department of Public Health and Sport Sciences, Faculty of Social and Health Sciences, Inland Norway University of Applied Sciences, Elverum, Norway; b Department of Education and Lifelong Learning, Norwegian University of Science and Technology, Trondheim, Norway; c Department of Health and Caring Science, Faculty of Health and Social Sciences, Western Norway University of Applied Sciences, Bergen, Norway

**Keywords:** Coping, development, serious mental illness, recovery, salutogenesis, qualitative research

## Abstract

**Purpose:**

The purpose of this study is to explore experiences with daily challenges and the development of salutogenic copings skills among young adults with serious mental illness.

**Methods:**

Nine young adults with serious mental illness were interviewed. The interviews were transcribed and subjected to reflexive thematic analysis.

**Results:**

Two main themes were identified through the analysis: “The influence of symptoms in everyday life and challenges with participating in the community” and “Making the small things matter.” The findings show that different life experiences, or on-going challenges, often can affect or interfere their lives on a daily basis, and that previous experiences with psychosis can contribute to the development of salutogenic coping skills.

**Conclusions:**

This study shows the importance of increased knowledge and awareness of different life experiences and challenges among people with serious mental illness such as schizophrenia spectrum disorders. It is significant to strengthen the ability to identify and use appropriate resistance resources to promote salutogenic coping skills and thus better health. Furthermore, it is important to be aware that the development of salutogenic coping skills must be personalized to cultural contexts and society as well as supported by local communities, families and services.

## Background

Serious mental illness (SMI) such as schizophrenia spectrum disorders are characterized by an extensive disruption of thinking, perceptions, and emotions. Thus, the person has severe challenges due to the stress of change in their experience and interpretation of reality (Davidson, 2020). These illnesses often occur in early adulthood and affect the person’s best years of work and productivity (Shrivastava & Sousa, 2020). The illnesses are also one of the major causes of disability among young people aged twenty to forty years (McGrath et al., 2008). Studies show that detection and treatment in the early stages of the illnesses clearly reduces symptoms and improves overall level of function (Melle et al., 2008). Torgalsbøen et al. (2018) found that the potential for improvement appears to be greatest in the first years after the onset of illness and that the ability to adapt and use available resources seems to be affected by the intensity and duration of the psychosis. In addition, it seems that persons who have achieved a certain level of progress in their recovery are more able to use and learn from the adversity of their psychosis as they seem to reflect on their experiences differently than those who experience less progress. Thus, it is important to identify the person’s ability to respond to the adversity from the illness, by grasping the person’s perception and thus tailoring treatment (Torgalsbøen et al., 2018).

In addition, a recent study concluded that the way a person appraises stress, the person’s level of resilience and the person’s sense of coherence are important factors that influence psychological well-being among people with SMI (Izydorczyk et al., 2019). Furthermore, when people appraise stressors as challenges, their sense of manageability is affected in a positive direction. Also, a person’s sense of comprehensibility is an important factor in the treatment and improvement of SMI, and it is vital to focus on helping the individual to enhance resilience (Izydorczyk et al., 2019).

A new report from the United Nations Special Rapporteur on the right of everyone to the enjoyment of the highest attainable standard of physical and mental health, Danius Pūras (2020), suggests a move in public health practice from a biomedical, risk-oriented discourse towards a focus on the settings of everyday life and links to human dignity and human rights. Health promotion is based on human rights and people-centred approaches, where citizens are placed at the heart through participatory and empowering processes (Lindström & Eriksson, 2006; Pūras, 2020). This notion is compatible with the theory of salutogenesis which is a health promotion theory (Antonovsky, 1996) that focuses on improving coping as a Sense of Coherence (SOC) and other coping resources. More than twenty years ago, Landsverk and Kane (1998) showed the benefits of a salutogenic approach in individuals with SMI to promote coping and health.

Despite that the importance of such an understanding and approach is well documented, it is rarely applied in research and in the current treatment of people with such conditions (Jormfeldt, 2011; Langeland & Vinje, 2017).

Although there are some studies that focus on health promotion among people with SMI; studies on SMI from a salutogenic health promotion perspective, are lacking. Thus, the main purpose of this study is to explore experiences with daily challenges among young adults suffering from SMI and how they cope with these challenges from a salutogenic perspective.

## Theoretical framework

The theory of salutogenesis focuses on what contributes to the development of good health and is a contradiction to pathogenesis, which explains disease. Through a salutogenic orientation, the important question is how a person can be supported to identify and use different resources thus promoting health, (Antonovsky, 1996). Antonovsky (1987, 1996) explored how people could move towards the health pole of the ease/dis-ease continuum, and he found that a level of sense of coherence (SOC) contributes to the level of health. By experiencing coherence in life, the world is perceived as more or less comprehensive, manageable, and meaningful. Comprehensibility refers to the degree to which the person feels that inner and outer stimuli are cognitively understandable and provide coherent information. Manageability refers to the extent to which an individual perceives that the resources available may be sufficient to deal with the situation. Meaningfulness refers to the degree to which the person experiences meaning in what is going on or wants to find meaning in what is happening These notions also imply that life stresses are a natural part of life that must be handled and that an individual is understood as participating in an active interaction with both inner and outer environments.

The importance of understanding and contributing to the person’s ability to adapt is fundamental in the salutogenic perspective (Antonovsky, 1987). Through active adaptation, a person has the opportunity to increase his/her SOC, well-being and health (Antonovsky, 1987; Langeland et al., 2016). As a prerequisite for dealing with a situation that is experienced as stressful, it is necessary to understand the situation to some extent (Eriksson & Mittelmark, 2017). A person’s SOC develops throughout life, and is especially affected of life experiences of consistency, load balance, participating in decision-making and emotional closeness (Eriksson & Mittelmark, 2017). According to Antonovsky (1996), it is more likely that a person with a higher SOC perceives stimuli from the environment as coherent and comprehensible than a person with a lower SOC. People who are chaotic and/or psychotic need support in finding order out chaos by focusing on such as comprehensibility, manageability and meaning, thus promoting SOC.

SOC is strengthened by experiencing appropriate challenges (balance between under- and overload) in daily life (Antonovsky, 1987; Langeland et al., 2007). Antonovsky (1987) emphasizes that even though a strong experience of manageability depends on a strong experience of comprehensibility, it seems as if the motivation factor within the meaningfulness component of SOC is the most important component. Further, without motivation, the person stops reacting to stimuli, and confusion occurs along with the lack of willingness to search for possible resources (Antonovsky, 1987).

Along with SOC, access to and use of various resistance resources are essential in the salutogenic model. In the same way that health can be seen as a continuum, Antonovsky emphasized that General Resistance Resources (GRRs) exist on a continuum from well-developed to poorly developed resistance resources (Antonovsky, 1987; Idan et al., 2017). GRRs may include such as social support, ego-identity, material resources, physical activity, coping strategies, knowledge and intelligence, and the absence or a lower level of these resources is defined as Generalized Resistance Deficits (GRDs). While GRRs contribute to experiences, which strengthens and preserve a strong SOC, GRDs act in the opposite manner and contribute to experiences which undermines SOC (Antonovsky, 1987). Another group of resistance resource involved are called specific resistance resources (SRRs), and these resources are more situation-specific resources (Mittelmark et al., 2017). Thus, both GRRs and GRDs contribute to the development of an individual’s SOC (Idan et al., 2017).

## Methods

### Design

In this study, a qualitative design was used to explore the participants lived experiences with daily challenges and the development of salutogenic coping skills among people with serious mental illnesses. The qualitative design focuses on the subjective experiences of individuals and groups, and illuminate lived experiences and to reveal meaning through a process of understanding and interpretation (Lindseth & Norberg, 2004; Van Manen, 2014).

To obtain the participants’ experiences, individual interviews were performed. The transcribed interview texts were subjected to reflexive thematic analysis as described in the literature (Braun & Clarke, 2006; Braun et al., 2014; Clarke & Braun, 2017; Braun & Clarke, 2020). Thematic analysis (TA) is a method for identifying, analysing, and interpreting patterns of meaning (“themes”) within qualitative data.

### Recruitment and participants

This study is a part of a follow-up study of the “Effects of Physical Activity in Psychosis” (EPHAPS) study (for further details see Andersen et al., 2018; Engh et al., 2015).

In the current qualitative study, recruitment was performed by strategic selection (Patton, 2002) of the 83 participants who had participated in the EPHAPS study. The participants were recruited based on the following inclusion criteria:

- Less than 30 years of age (younger adults were the main target group) when participating in EPHAPS.
- Considered to be in their habitual state and understood the purpose of the study.
- Participated in the exercise group (This criteria was applied due to another part of the follow-up study that explores physical activity and mental health).

The first author and researchers in EPHAPS jointly discussed which participants from the EPHAPS study that could be relevant participants in the qualitative follow-up study based on experiences from conducted interviews and surveys in EPHAPS. It was considered essential that the participants had to be able to give rich descriptions of their own experiences and would not experience participation in the follow-up study as an additional burden.

Current or last known therapists were then contacted to discuss whether the participant could be contacted regarding participation in the follow-up study. This was an ethical demand from the hospital management to prevent overload among possible participants and was thus performed to minimize the risk of possible negative consequences from participating in the study.

In agreement with the therapists, 14 relevant participants were contacted by telephone and given information about the study and the possibility of participation. During two of the telephone conversations, it became clear that these persons were not in their habitual state and were therefore not eligible for participation. Two replied that they wanted to participate after receiving information about the study over the phone. However, they did not respond to later inquiries when more formal informational talks were to be conducted despite repeated attempts to make contact. One withdrew after signing the consent due to fear that participation in the study could lead to the worsening of symptoms. Thus, nine participants (three woman and six men) finally agreed to participate. Seven of the participants were unemployed and received state benefits. One received state benefits and worked in a municipal work initiative a few hours a day, and one had a regular paying job. Due to anonymity, further details are omitted. Prior to the interview the first author and the participant met and the information about the study was given again to ensure that the participants understood the aim and type of study.

### Research ethics

The study was approved by Regional Ethics Committee of Southern and Eastern Norway (REC number 2014/372) and complies with the principles of the Helsinki Declaration.

In medical research, there is a special requirement to execute caution when conducting research with vulnerable groups.

To protect the participants, prior to the in-depth interviews, it was verbally agreed that the interviewer (first author) could inform the participant’s contact person or GP (general practitioner) if it emerged during the interview that the participant was in a phase that required increased follow-up. None of the participants were in a condition that necessitated that the interviewer contact the therapist or GP. One of the participants was voluntarily admitted to a sub-acute facility due to a mild depression, and the conduct of the interview was therefore clarified with the therapist and was subsequently divided over two days to minimize strain.

The participants were given thorough information about the study both verbally and in writing to ensure informed consent. They were also informed of possible benefits and risks and that they could withdraw from the study at any point. None of the participants withdrew their consent. For those who wanted to extend the interview over two days, the time for the second part of the interview was agreed upon at the end of the first interview. Between interviews, the participants received “treatment as usual” depending on the follow-up they typically received. During the in-depth interviews, the interviewer was aware of signs that could indicate that the participants were feeling uncomfortable or did not want to talk about the subject. If this occurred, the interviewer asked if the subject was too difficult. In some cases, it was necessary to change the subject to avoid the participant from experiencing unnecessary strain. To safeguard anonymity and protect the participants’ identities, details such as names and places have been changed. All names used are pseudonyms to ensure the participants’ anonymity.

### Data collection: qualitative interviews

We used individual qualitative in-depth interviews to collect data. A semi-structured interview guide was developed based on the topics of the research questions. The interview guide was reviewed and edited by the first author and two participants from the main study who met the criteria regarding diagnosis, age, etc. but did not participate in this sub-study. This procedure was implemented to ensure that the interview questions were as accurate as possible and simultaneously understandable to the participants.

The interview guide was developed with open-ended questions that addressed the participants’ experiences and views on the various topics. The overall interview questions used were: *Can you describe how psychosis experiences affect or have affected you? Do you feel that these experiences have led to some sort of increased insight or better understanding? What do you experience as meaningful in your everyday life?*

The first question served to invite the participant to tell about their life in general and the two others were more specified to get specifically data about salutogenesis.

The first author conducted all of the in-depths interviews, and each interview lasted between 90 and 150 minutes. The interviews were audiotaped and transcribed verbatim by the first author.

The interviews were performed between July 2019 and November 2019, and were conducted in agreement with the patients on premises of the Division of Mental Health and Addiction, Vestfold Hospital Trust in Norway and in agreement with the participants’ therapists. To provide a feeling of security, 45–60 minutes were spent prior to the interview to get to know each other and create a safe atmosphere. All participants were offered the opportunity to have the interviews conducted at their home if desired. The last half of one of the interviews was conducted at the home of the participant at his/her request. To ensure that the participants were understood correctly, their answers were summarized and returned to the participants to check that their descriptions and views were understood correctly.

### Data analysis

A thematic analysis to explore and understand the participants’ lived experiences was applied (Braun & Clarke, 2006; Braun et al., 2014). The following steps was used:

1. The first step of the analysis consisted of becoming familiar with the data by reading and re-reading through of all transcribed interviews to obtain an overview of the lived and subjective experiences of the participants, noting initial thoughts, ideas, and emerging themes.
2. Subsequently, each interview was thoroughly reviewed, and initial ideas or meaningful units that were considered relevant to the research question were identified and initially coded. The first two steps were performed individually by the first, second and fourth author.
3. The initial coded ideas and emerging patterns of the participants lived experiences were identified, condensed, interpreted, and categorized into potential patterns (themes) across the participants by the first author.
4. Meaningful elements, such as quotes and descriptions of the emerging themes, were identified, collated, and then sorted into four tentative main categories (experience of meaning in everyday life, mastery, coping strategies, and influence of psychosis symptoms). Furthermore, these meaningful units were systematized using NVivo (version 12), and various meaning units were categorized into different themes. Several of the meaning units had meaning content that suited different themes, and a meaningful entity could therefore be categorized into several themes or sub-themes. This step was performed through an analysis seminar by the first, second and fourth author.
5. Subsequently, the first, second and fourth author jointly discussed the relevance of the potential themes ensuring that they were focusing on the lived experiences of the participants. This information was reviewed regarding internal homogeneity and external heterogeneity, and similarity between themes was examined. Overarching themes were identified, and a thematic map was generated to show coherent connections and differences between the themes and sub-themes.
6. To ensure internal validity, the second, third and fourth authors discussed the process of analysis and the findings with the first author. They also contributed to writing the text describing the themes and the ensuing discussion. To reduce the risk of research bias, preliminary analysis steps were discussed jointly to identify similarities and differences in prominent opinion content, themes, and sub-themes. Finally, the discussions resulted in more precise themes and sub-themes that captured the descriptions and experiences that emerged in the interviews. Through the process of the thematic analysis, we identified two overarching themes responding to the participants lived experiences with daily challenges and the development of salutogenic copings skills among young adults suffering from SMI; (1) *The influence of symptoms in everyday life and challenges with participating in the community*, and (2) *Making the small things matter*. See Table I for an example of the process of analysis.

**Table I.** Example of the process of analysis

Meaningful unit	Code	Subtheme	Main theme
“It affects me a great deal. It keeps me from getting out. Prevents me from being with friends. Prevents me from doing things I want to do.” “I often recognize when a psychosis begins. I can feel now this is happening and maybe that will be happening. But, if I do this or maybe that, then it can calm down.”	Influence of psychosis symptoms	To be “owned” by the illness Obstacles to participation Use of experience	The influence of symptoms in everyday life and challenges with participating in the community
“And look at what I manage, which is not the biggest thing. Or, I *can* do a lot of big things, but what I actually manage are the smaller things in a way.”	Experience meaning in everyday life	Searching for meaning Reward yourself	Making the small things matter

## Results

In this section, we will present the results from the thematic analysis regarding the challenges that young adults with SMI experiences in their everyday life and how those challenges influence their coping skills.

### The influence of symptoms in everyday life and challenges with participating in the community

The participants talked about the influence of symptoms in their everyday life and challenges with participating in the community. Several of the participants described that they felt affected by different types of psychotic symptoms during the day, which could make participating in social life very challenging. The symptoms could be experienced as an obstacle to being with friends or performing activities as planned, and these obstacles often related to anxiety or confusion caused by the symptoms. The aspects of this overarching theme that will be described are (1) *to be “owned” by the illness*, (2) *obstacles to participate*, and (3) *use of experience*.

#### To be “owned” by the illness

The participants described how the presence of different symptoms was often impossible to ignore and often resulted in a sense of vulnerability and a need to protect oneself.

The influence of symptoms could be very strong and impossible to ignore as the participant Siri described:

*“I get a little caught by the disease, so I kind of forget that I manage to be healthy or better—somehow. And then it becomes a thing of aggravation*. *And in a way, everything blackens—even more.”*

Different types of symptoms, such as paranoia, anxiety, insecurity, extended self-focus and mental changes with blackouts, resulted in changes in various ways and could influence participation on a daily basis. Participants such as Frank described how it feels to have loud sounds in your head that you cannot mute:

*“It’s kind of very close, like most of the time. But when it gets bad, is it extremely loud! So, then it will be in a way that you will not be able to mute. You can’t focus on anything other than the voice. I have not found a way to solve it*.*I try to sleep, actually. I listen to loud music. I am kind of trying to focus, to somehow get away—do something else.”*

Ann also experienced sounds in her head and extensive paranoia, and she expressed the experience as follows:

*“Sometimes I just hear it as a whispering, but sometimes it is like a big roar inside my ear somehow. Sometimes I feel like I have to wear sunglasses, because I feel that people look straight into my soul somehow.”*

The feeling of people being able to see into one’s soul and the need to avoid this was also described by Ole:

“*I have often worn sunglasses. Because, then people don’t see me in a way*. *I felt I was like an open book for anyone who met me.”*

Espen described how he only managed to focus on himself when the illness and the anxiety deepened:

*“Especially when I feel insecure, when I feel scared and I feel … It is very often these thoughts - that in a way - that comes, they come very brutally. Then, you just focus on yourself and your place in the world. This means that you cannot get other perspectives and see the context.”*

#### Obstacles to participate

The participants described that the symptoms made it very difficult to participate in school and obtain an education. Eva described what it feels like when one must drop out of school and forego education because of an illness such as this:

“*I thought I would graduate from high school with really good grades. And somehow get a good education and a job … But then I did not manage after all – I dropped out.”*

In the group of participants, those who dropped out of school were young people with hopes of “really good grades” as Eva described above. Frank, who was disturbed by loud sounds in his head on a daily basis, had a similar experience and felt that the symptoms were very exhausting and made it difficult to participate:

*“I’m getting tired, very tired afterwards. It is really exhausting. Maybe, I am getting a little darker too, by just being … I have been trying to go to school but … I have gone to high school. But I did not complete anything there either.”*

Trond explained that his illness and the symptoms made it very challenging to participate in the community:

*“It affects me a great deal. It keeps me from getting out. Prevents me from being with friends. Prevents me from doing things I want to do.”*

When Per felt influenced by symptoms, he felt that he should be alone until he felt less overwhelmed:

*“Then, I choose to stay at home and relax, just relax properly. Try to be alone for one day. The hardest thing is that I just sit at home and know that I can’t do anything else for the rest of the day - in a way.”*

#### Use of experience

The participants experienced psychotic influences as predominantly negative events that caused great difficulties in their lives. Nevertheless, they also indicated that these experiences contributed to a better ability to recognize when symptoms emerged and thereby reduce some of the stress in the situation. When Trond felt that he was influenced by symptoms, he tried to focus on being in reality and convince himself that what was happening was not real:

*“Sounds or things I see. I try to think that this is a misinterpretation - this is not true. Something like that I try to think. And when I manage to think like that, it actually helps quite a bit in that situation. But I don’t always manage to pull it off.”*

The identification of a possible illness symptoms based on previous experiences was also useful, as described by Eva:

“*I often recognize when a psychosis begins. I can feel now this is happening and maybe that will be happening. But, if I do this or maybe that, then it can calm down.”*

Similar experiences were also emphasized by Ole, who said the following:

*“It has made me a stronger person. It has given me the opportunity to sense when a psychosis begins to arise. And I have gradually learned to see reality and fantasy*
*in two different ways—instead of being merged.”*

When Erik started to feel influenced by symptoms, he could cope with the situation by moving his focus away from the symptoms and re-thinking the situation. He expressed this as follows:

*“I know in a way what I need to do to get better. I often move focus to get away from the situation in a way, to feel that one is doing something that matters.”*

The participants described that they often experience psychosis symptoms as very intense and close. Furthermore, the symptoms feel unhealthy and can lead to difficulties in considering different perspectives and focusing only on themselves. Some also noted that the symptoms can become so overwhelming during the worst period that they experience black outs. Although the psychosis experiences were perceived as predominantly negative, the participants also felt that they could use knowledge from previous psychosis experiences to understand what was happening and thus be more able to cope with the situation.

### Making the small things matter

Most of the participants were out of work and received disability benefits. Failure to participate in work or schooling appeared to be experienced as a defeat, and the experience of inadequacy emerged from the interviews with many of the participants. Not being able to meet expectations, such as getting an education or being employed, appeared to be a source of a feeling of being less valued as a person and provided an experience of vulnerability and hopelessness. In order to manage this difficult life situation, the participants indicated that it was important for them to make the small things matter in their everyday life. The aspects of this overarching theme that will be described are (1) *searching for meaning*, and (2) *reward yourself*.

#### Searching for meaning

Several of the participants struggled to fill their days with activities that provided a sense of meaning, but they tried to find satisfaction in the things they actually did. Trond said that he struggled to find meaning and initiative to do other things besides computer games and that almost everything felt challenging. This feeling was expressed by the following statement:

*“Computer games are what keep me going, I am sure of that. When I manage to do things that are difficult, I gain mastery and motivation- in example to sit on the porch outside.”*

Several of the participations, such as Siri, Anne and Eva, underlined contact and cuddling as well as responsibility for dogs or cats as curative, indicating that these activities improved their lives. The possible impact of contact with an animal is expressed by the following statement by Eva:

*“My dog is the most important thing … because she must be taken care of …* .
*She needs food and a walk. She must have attention; she must have training; she needs to play.”*

In relation to the same experience, Siri said the following:

*“I get out with the dog, and in a way, because then it’s easier not to drag himself down then.”*

Eva and Espen talked about cleaning and making their own food as helpful ways to have a meaningful life. Eva expressed this sentiment as follows:

“*I am so fond of doing housework. It is very okay to cook, clean and re-furnish a little.”*

Espen also expressed that these activities were important:

*“Not that it should be something comfortable—that I want to do it*.
*But that I do it, because it is necessary or that it is something that I enjoy.”*

These quotes indicate that the participants experience some benefit from having animals in their lives and that they feel responsible for their animals. More trivial things, such as doing housework, could also contribute to a sense of meaning in everyday life because it is something that is necessary, even if it is not necessarily fun. These examples of meaningful activities underline that people with SMI search for the same experiences that also give meaning for people in general and are thus not experiences unique for them.

#### Reward yourself

The importance of trying to lower expectations and find a sense of satisfaction in what one actually manages to achieve was emphasized by several of participants.

Espen emphasized the importance of both the initiative to do small things and the importance of caring for oneself instead of focusing on other people:

*“Being able to cope with challenges, being able to work for oneself*.
*That you take some initiative in relation to maybe doing small things for yourself and not for everyone else.”*

Siri expressed thoughts similar to those reported by Espen regarding the importance of appreciating the things she managed to do, even if they seemed trivial:

*“And look at what I manage, which is not the biggest thing. Or, I can do a lot of big things, but what I actually manage are the smaller things in a way …*
*But if I look at the little things I do, I lift myself up.”*

To summarize, the participants experienced several different challenges and obstacles in their lives, and as described, they often had different methods for solving them. The participants focused on coping with small things in their everyday life, such as walking the dog or doing housework. They rewarded themselves for what they actually managed and tried to create a sense of meaningfulness in their life.

## Discussion

This study used a salutogenic perspective to understand the lived experiences of young people with SMI and how they coped with their daily challenges.

### The importance of appropriate challenges

As our results show, the presence of symptoms made it difficult for the participants to connect with others and participate in “real” life, and the presence of symptoms could cause confusion, insecurity, paranoia, anxiety and extended self-focus. The experience of psychotic symptoms can be interpreted as a type of disturbance in correctly perceiving inner and outer stimuli (Davidson, 2020), thus experience overload and chaos. According to the theory of salutogenesis (Eriksson & Mittelmark, 2017), stimuli received from inner or external environments must make some sort of sense and provide coherent information for a person to experience comprehensibility. Experiencing different symptoms often caused difficulties comprehending situations and experiences of inconsistency in what is occurring, which seemed to lead to overload and lack of general and/or specific (GRRs and/or SRRs) to cope with the situation. The importance of comprehensibility was also demonstrated in the study of Izydorczyk et al. (2019), which emphasized that the comprehensibility and manageability component of SOC is of great importance in achieving improvement for people with SMI.

As our results show, an attempt to manage the disturbance caused by the symptoms by changing focus to something more substantial or tangible was useful. By focusing on something else that felt important, several participants reported that their symptoms disappeared or were less intense. Some stayed at home as a strategy because they had experienced that their symptoms disappeared if they stayed home for a while even though it felt frustrating, as they could not do anything other than wait. Others tried to sleep or listen to loud music in an attempt to change focus and cope with the stress caused by their symptoms. Such ways of coping with these specific challenging situations might be seen as active adaptation to difficult situations and might function as SRRs.

According to salutogenic theory, a person’s ability to actively adapt to situations can be essential to coping with stressors. This means that it is important to balance between under- and overload, thus improving coping abilities (Antonovsky, 1996). Langeland et al. (2007) also underline that SOC is strengthened by experiencing appropriate challenges (balance between under- and overload) in everyday life. Several of the participants sensed when symptoms were starting to arise and had experienced that a “reality check” based on similar previous experiences was useful to try to better comprehend the situation. For several of the participants in our study, this strategy was useful and contributed to a more successful management of the situation, thus developing a salutogenic coping skill. Similar findings were also reported in the study by Torgalsbøen et al. (2018) who found that people who show significant recovery from the disease, seems to learn and reflect on previous experiences from psychosis and thus being able to adaption and use of available resources in management of stressors. This is compatible with the theory of salutogenesis where a main aim is that the person come into a positive interplay between SOC and use of GRRs/SSRs, thus promoting the transformation of tension into salutogenic coping (Langeland et al., 2016; Mittelmark et al., 2017). Following this theory, the use of knowledge from previous experiences to cope with a specific stressor, such as psychotic influences, can therefore be seen as the use of SRR, thus promoting SOC.

However, for some participants, this strategy did not work or was not used. Based on how the participants appraised and experienced the tension caused by symptoms, it appears that some appraised the stressor as overwhelming and a type of threat. This finding might be explained by differences in the intensity or type of symptoms and overload, but it might also indicate a lower SOC and GRDs and lack of motivation to cope with the situation. The study by Izydorczyk et al. (2019) also found similar findings and showed that stressors appraised as threats causes negative emotions and negatively affect the person’s management of stressors.

In the study by Torgalsbøen et al. (2018) it was found that the intensity and duration of the psychosis appear to affect the ability to adapt and use available resources. Furthermore, those who are non- recovered are less capable to reflect and learn from the adversity of previous psychotic experiences.

Our results show that the presence of symptoms and difficulty ignoring or relating to them in a way that promotes health may indicate that a person is unable to understand a situation and accordingly does not have the resources to create order out of chaos, thus experiencing overload. Antonovsky (1987) argues that a strong SOC can contribute to better use of available and appropriate GRR and SRR thus experiencing appropriate challenges and subsequent movement towards health. Disturbances in especially comprehensibility as a result of the influence of symptoms will probably have a direct impact on both the SOC and the GRRs-/GRDs interplay (Antonovsky, 1987; Idan et al., 2017).

### The significance of meaning in life

The need for some type of reorientation of their own expectations and experiences seemed to be consistent among the participants, highlighting the importance of appreciating the smaller things in life, thus promoting meaning. Experiences of not being an active part of one’s life and instead being a “passenger” who has little or no influence on one’s life events were evident among the participants. Participation trough making decisions about one’s own life is essential for people to experience dignity and control in life and allows individuals to be involved in the community (Ness, 2016). As part of coping with everyday challenges and promoting participation in the community, our results showed that small things matter. Davidson and Johnson (2013) argue that managing seemingly smaller things can provide a sense belonging in one’s community and strengthening a sense of self. Further, that without a sense of self there is no motivation to try to counteract the adversity from the illness (Davidson & Johnson, 2013). Focusing on small affirmations from others can contribute to a sense of identity as a valuable member of society. Thus, seemingly small things can promote a stronger sense of self that is more stable and can withstand adversity caused by the illness (Davidson, 2020). The value of knowledge about how small things can promote health is essential to improve the quality of care when supporting people with SMI (Topor et al., 2018). Ness (2016) also emphasizes the value of small things, but also points out that people sometimes need more tangible support, and that more practical help and support may be needed.

According to salutogenesis, active involvement in decisions regarding one’s own life is important and contributes to the basis of the meaningfulness component of SOC (Eriksson & Mittelmark, 2017). By actively focusing on seemingly smaller things, the participants became more able to create a load balance that reduced the experience of stress, simultaneously as it increased the feeling of well-being. The value of smaller things is emphasized by Davidson and Johnson (2013), Davidson (2020), and Topor et al. (2018) through providing hope, strengthening one sense of self and not feeling reduced to a diagnosis. Furthermore, that a stronger sense of self contributes to having the necessary motivation to try to counteract the adversity from the illness as underlined by Davidson and Johnson (2013).

This finding is consistent with Keyes (2014), who emphasized that good mental health and challenges in life can be present over the same period of time. From a salutogenetic perspective, focusing on managing the smaller things in life can contribute to a better experience regarding load balance, meaning and thus a stronger SOC and a movement towards better health and thus possibly reduce the symptoms of mental illness

The participants in our study described the search for meaning in life as challenging. For some, this led to extended focus on things that provided a sense of meaningfulness, such as computer games and housework. Focusing on their pets lead to a sense of responsibility and increased well-being. Further, this focus and feeling of responsibility felt meaningful and occasionally seemed to be a coping strategy that led to a sense of mastery over and distraction from difficult situations, e.g., by walking the dog. Focusing on the smaller things led to an understanding of the importance of doing things for oneself and not for others. This can be understood as a more active engagement in one’s life and leads to an increased sense of participation. Such active participation is seen as essential in salutogenic theory, because it is the basis of the meaningfulness component of SOC (Eriksson & Mittelmark, 2017).

Based on our results, we argue that it is important to identify or help to identify the various challenges, thoughts and understandings a person has regarding resistance resources and the context of his/her life to promote health or help the person move towards the health pole on the continuum. Izydorczyk et al. (2019) noted that a sense of comprehensibility is significant for improvement and that treatment should focus on supporting the person in enhancing mental toughness and trying to appraise stressors as challenges rather than threats.

However, from a salutogenic perspective, people are always in mutual interplay with their environment. Salutogenic coping skills are always created in this interplay. Thus, it is crucial that people with SMI are seen in cultural contexts and as part of the society and are supported by communities, families and services (Davidson, 2020).

### Limitations and strengths of the study

One limitation is that the sample consisted of only nine participants, and more participants could have increased the variety of the descriptions and experiences. However, one strength is that the in-depth interviews lasted up to two and one-half hours, and gave the participants sufficient time to give thoughtful and thorough descriptions. Breaks were also included when necessary. Another strength is that interviews with some of the participants were conducted over two days/two meetings depending on the participants’ health condition on the interview day. This format ensured that the participants were able to provide comprehensive and rich descriptions of their experiences. Several of the participants had met the interviewer in the role of an employee in the EPHAPS study when they participated in that study. This familiarity promoted a sense of safety during the interview. Further, the interviewer’s many years of experience as a psychiatric nurse in the mental health care field and especially with this vulnerable group may also have contributed to an increased sense of security and thus created an open environment for sharing experiences and perspectives.

### Conclusions and implications for practice and future research

The present study shows that people with SMI have different challenging life experiences that affect or interfere with their lives on a daily basis. Our study also reveals that these individuals develop different salutogenic coping skills when facing these challenges.

The study shows that focusing on seemingly smaller, but meaningful things can reduce stress and increase one’s feelings of a sense of self, thus creating the necessary motivation to cope with the challenges caused by the illness.This finding underlines the significance of developing knowledge about the challenges people with SMI face from a salutogenic perspective because we then increase the knowledge about how these people use their resources to make order out of chaos, thus possibly promoting their SOC. Further, such knowledge is likely important to support these people in developing coping skills, meaning and thus better health. Such knowledge is also important in order to be able to improve existing care, thus provide a more tailored treatment that focuses on increasing salutogenic coping skills, active participation and strengthening of the SOC. However, it is necessary to perform several larger such studies or repeating our study as a longitudinal study to develop increased knowledge from a salutogenic perspective about people struggling with SMI.
